# A novel hybrid MCDM framework combining TOPSIS, PROMETHEE II, and VIKOR for peach drying method selection

**DOI:** 10.1016/j.crfs.2025.101034

**Published:** 2025-03-16

**Authors:** Burak Gülmez

**Affiliations:** a Department of Industrial Engineering, Mudanya University, 16940, Mudanya, Bursa, Türkiye; b Leiden Institute of Advanced Computer Science, Leiden University, Leiden, The Netherlands

**Keywords:** Peach drying technology, Multi-criteria decision-making, TOPSIS, VIKOR, PROMETHEE II, Food processing optimization, Sensitivity analysis, Technology selection

## Abstract

The selection of optimal drying technologies for peach processing presents a complex decision-making challenge due to multiple conflicting criteria. This study introduces a novel hybrid multi-criteria decision-making (MCDM) framework combining TOPSIS, VIKOR, and PROMETHEE II methods to evaluate eight drying technologies. The evaluation was conducted across twelve criteria, encompassing product quality, operational efficiency, economic factors, and environmental impact. Data were collected from five industry experts through structured matrices. The results demonstrate that vacuum drying emerged as the optimal technology, maintaining the top position in 75 % of sensitivity scenarios. Freeze drying and heat pump drying consistently ranked among the top three alternatives across all methods. The correlation analysis revealed strong agreement between VIKOR and PROMETHEE II rankings (0.857), while TOPSIS provided complementary insights. Sensitivity analysis identified energy consumption, investment cost, and nutritional retention as the most critical factors influencing technology selection. The findings indicate that advanced drying technologies significantly outperform traditional methods in terms of overall performance. This research provides a comprehensive framework for evidence-based decision-making in food processing technology selection and establishes quantitative benchmarks for future technology evaluations in the fruit drying industry.

## Highlights

• Novel hybrid MCDM framework integrates TOPSIS, VIKOR, and PROMETHEE II for peach drying selection.
• Vacuum drying emerges as optimal technology, maintaining top position in 75 % of sensitivity cases.
• Expert-based evaluation across 12 criteria validates advanced drying methods over traditional ones.
• Monte Carlo simulation with 1000 iterations confirms framework reliability and result robustness.
• Energy consumption, investment cost, and nutritional retention identified as critical factors.

## Graphical abstract

**Figure:**
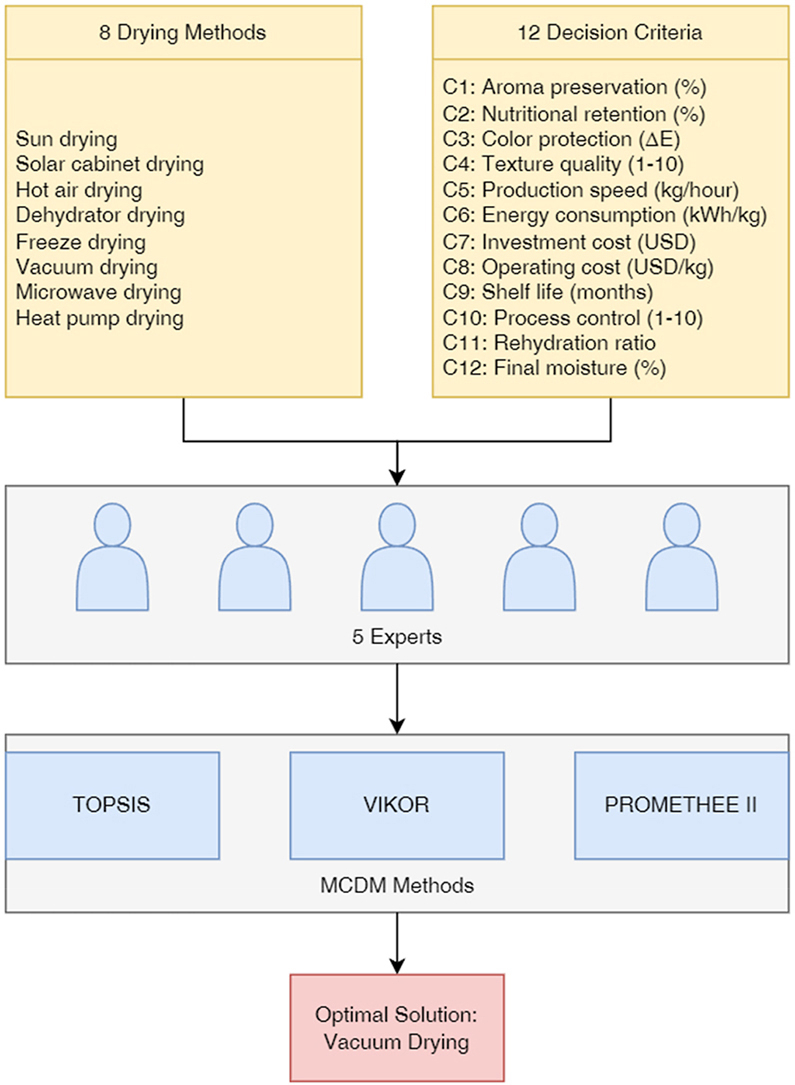

## 1 Introduction

Peaches (Prunus persica) are a popular stone fruit known for their sweet flavor, juicy texture, and vibrant color. They belong to the Rosaceae family and are cultivated in temperate regions worldwide. Peaches are not only enjoyed fresh but are also processed into various products, including dried fruits, which have gained significant popularity due to their convenience and extended shelf life. The global peach production has been substantial, with countries like China, the United States, and Italy leading in both production and export. In 2021, global peach production was estimated at approximately 25 million metric tons, with China accounting for over 60 % of this total, followed by the United States and Italy, which contribute significantly to the global market (Tan et al., 2022).

The drying of peaches is a critical process that enhances their shelf life and preserves their nutritional and sensory qualities. Dried peaches are rich in vitamins, minerals, and dietary fiber, making them a healthy snack option. The global dried fruit market is projected to grow significantly, with dried peaches being a notable segment. In 2020, the dried fruit market was valued at approximately USD 8 billion, with a compound annual growth rate (CAGR) of 5.5 % expected through 2027 (Doymaz, 2014). The economic importance of peaches extends beyond their consumption; they are also vital in agricultural economies, providing income and employment opportunities in rural areas.

The drying process of peaches can be accomplished through various methods, including sun drying, hot air drying, freeze drying, and microwave drying, among others. Each method has its advantages and disadvantages, affecting the quality attributes of the final product, such as aroma preservation, nutritional retention, color protection, and texture quality. For instance, studies have shown that hot air drying can lead to significant losses in anthocyanin content, which is crucial for the fruit's color and health benefits (Lyu et al., 2015). The selection of an appropriate drying method is essential to optimize these quality attributes while considering factors such as production speed, energy consumption, investment and operating costs, and shelf life.

The significance of selecting the right drying method cannot be overstated, as it directly impacts the quality and marketability of dried peaches. The choice of drying technique influences not only the sensory characteristics of the product but also its nutritional profile and economic viability. For instance, freeze drying is known for preserving the nutritional content and flavor of fruits but is often more expensive compared to conventional methods like hot air drying (Chatzilia et al., 2023). Therefore, a comprehensive analysis of various drying methods is necessary to identify the most suitable option for peach processing.

The novelty of this research lies in the systematic integration of three complementary MCDM methods to address the multifaceted challenges of drying technology selection. While previous studies have applied individual MCDM methods to food processing technology selection or combined two methods, the integration of TOPSIS, VIKOR, and PROMETHEE II specifically for peach drying technology evaluation represents a novel approach. Each method contributes unique analytical strengths: TOPSIS excels in handling quantitative criteria through distance-based evaluation, VIKOR provides robust compromise solutions when conflicting criteria exist, and PROMETHEE II offers sophisticated preference modeling through pairwise comparisons. This tripartite approach was deliberately chosen to overcome the limitations inherent in single-method analyses. Some studies have demonstrated that different MCDM methods can produce varying rankings when applied to identical problems due to their underlying mathematical principles. By employing multiple methods and synthesizing their results, this research mitigates method-specific biases and enhances decision reliability. The ensemble approach provides decision-makers with greater confidence in technology selection by identifying solutions that perform consistently well across different evaluation paradigms, rather than those that may be artificially favored by a particular mathematical formulation.

The primary objective of this research paper is to conduct a MCDM using an integrated TOPSIS-VIKOR-PROMETHEE II approach to evaluate and compare different peach drying methods based on multiple criteria. The criteria for evaluation include aroma preservation, nutritional retention, color protection, texture quality, production speed, energy consumption, investment cost, operating cost, shelf life, process control, rehydration ratio, and final moisture content. The alternatives considered in this study are sun drying, solar cabinet drying, hot air drying, dehydrator drying, freeze drying, vacuum drying, microwave drying, and heat pump drying. By employing this integrated approach, the research aims to provide a systematic framework for decision-makers in the peach processing industry to select the most effective drying method that aligns with their operational goals and quality standards.

## 2 Literature review

Tan et al. (2022) investigated the drying kinetics and anthocyanin degradation of blood-flesh peaches under hot air drying for the first time. They found that the drying process was characterized as reduced-speed drying, with the Page model effectively predicting moisture changes. The study revealed that while the total monomeric anthocyanin content decreased with higher drying temperatures, the optimal temperature for maintaining fruit quality was identified as 70 °C. This research provides valuable insights into anthocyanin behavior during drying, aiding in the production of high-quality dried peach products.

Wang et al. (2024a) investigated the effects of osmotic dehydration combined with vacuum freeze-drying on the characteristic aroma components of peach slices. They established a calibration curve for the aroma compounds using methanol as a solvent, which allowed for the precise measurement of vaporized compounds at varying concentrations. This methodology aimed to enhance the understanding of how different drying treatments affect the aroma profile of dried peaches.

Guo et al. (2022) optimized a pressurized liquid extraction (PLE) method to evaluate the neuroprotective potential of thinned peaches dried under various conditions. They characterized the PLE extracts using HPLC-Q-TOF-MS/MS and assessed their ability to cross the blood-brain barrier (BBB). The study found that freeze-dried samples extracted with 50 % ethanol at 180 °C exhibited the highest neuroprotective potential, highlighting specific metabolites that could contribute to Alzheimer's disease prevention.

Akhoundzadeh Yamchi et al. (2022) investigated the effects of high-power ultrasound pretreatment on the drying kinetics and physio-mechanical characteristics of peach slices. They found that ultrasound treatment significantly reduced drying time by up to 40 % and influenced the elastic modulus of the samples, decreasing it by approximately 46 %. The study also modeled the drying process using mathematical equations, concluding that ultrasound pretreatment is a promising method for enhancing the quality of dried peach products while requiring further research to optimize its application in food processing.

Lyu et al. (2015) conducted a comprehensive quality evaluation of yellow peach chips produced through explosion puffing drying. They analyzed nineteen evaluation indicators, including color, rehydration ratio, and texture, using various analytical methods such as principal component analysis (PCA), analytic hierarchy process (AHP), K-means clustering (KC), and discriminant analysis (DA). The study revealed significant variations in the quality indicators among different yellow peach cultivars, as indicated by the dispersed coefficient of variation ranging from 3.58 % to 852.89 %.

Inglese et al. (2002) investigated the effect of crop load on dry matter partitioning in peach trees of early and late ripening cultivars grafted onto different rootstocks. They conducted a two-year study involving destructive sampling of trees to analyze dry matter and carbohydrate distribution, revealing that increased crop load negatively impacted vegetative and root growth while influencing the allocation of dry matter to fruit. The findings highlighted the relationship between rootstock vigor, crop load, and overall tree growth dynamics, providing insights into optimizing peach cultivation practices.

Zhang et al. (2017) investigated the effects of osmotic dehydration (OD) and ultrasound-assisted osmotic dehydration (ULOD) on the drying kinetics and quality attributes of peach cylinders during infrared radiation drying. They also examined the moisture state and redistribution following osmotic pretreatments, utilizing low field nuclear magnetic resonance to analyze the changes in water loss and solute gain, finding that both osmotic time and ULOD enhanced water transfer.

Polat et al. (2021) conducted a study to evaluate the effectiveness of various drying techniques on peach puree. They implemented six different drying methods, including convective drying (CD), microwave drying (MW1, MW2, and MW3), and combined convective-pulsed microwave drying (CD + MW2 and CD + MW3). The study assessed the impact of these drying methods on several parameters, such as drying time, color, pH, Brix, and microstructural characteristics, concluding that convective drying resulted in the longest total drying time of 220 min.

Kingsly et al. (2007) investigated the effects of pretreatments (potassium metabisulphite and ascorbic acid) and drying air temperatures (55 and 65 °C) on the drying behavior of peach slices using a cross-flow tunnel dryer. They found that pretreatment significantly increased the drying rate of the peach slices, and the logarithmic model was identified as the most suitable for describing the drying behavior, demonstrating high correlation coefficients. Additionally, the effective moisture diffusivity was greater in the treated samples compared to the untreated ones, indicating enhanced drying efficiency.

Polat (2024) conducted a study to analyze the drying characteristics and quality attributes of peach slices using three different drying methods: electrohydrodynamic (EHD), hot air, and a combination of EHD and hot air (EHD-hot air). They evaluated the drying times, color, rehydration capacity, and microstructure of the dried peaches under varying conditions, specifically two air temperatures (50 and 55 °C) and electric field strengths (6.67 and 10 kV), while maintaining a constant air velocity. The results indicated that the EHD method resulted in the longest drying times, followed by hot air, with the EHD-hot air method demonstrating the shortest drying durations.

Zang et al. (2024) investigated the effects of combining ultrasound and ultra-high pressure as pretreatments to enhance the radio frequency vacuum drying of peach slices. They evaluated the impact of these pretreatments on drying characteristics, physicochemical quality, texture, and sensory attributes of the dried peach slices. Additionally, they assessed the free radical scavenging capacity of the peach slice extracts using the DPPH method, indicating a focus on both quality enhancement and health-related properties of the dried product.

Largo-Avila et al. (2024) investigated the effects of different drying methods on the physicochemical and sensory properties of surplus peaches (Prunus persica L.) in Colombia. They compared Refractance Window Drying (RWD) at various conditions, including RWD with maltodextrin, against conventional oven drying. The study found that the drying method significantly influenced the texture, color, and taste of the peaches, with RWD showing superior results in moisture reduction and sensory acceptability after storage. The findings highlight the potential of RWD as an effective drying technique for enhancing the quality and shelf life of peach products.

Barforoosh et al. (2024) investigated the drying kinetics of Haj Kazemi peach slices using a thin-layer dryer, examining the effects of various temperature levels, air velocities, and slice thicknesses on moisture changes. They employed an Adaptive Neuro Fuzzy Inference System (ANFIS) to model and predict the drying process, concluding that slice thickness significantly influenced drying time more than air velocity.

Doymaz and Bilici (2014) investigated the impact of citric acid pretreatment on the drying characteristics of peach slices using a cabinet dryer. They conducted experiments at various temperatures (45, 55, 65, and 75 °C) and found that the drying time for peach slices treated with citric acid was significantly shorter compared to untreated control samples.

García-Moreira et al. (2023) investigated the effects of different drying methods natural and forced convection at varying temperatures (40 °C, 45 °C, and 50 °C) and solar drying at two air velocities (1 m/s and 3 m/s) on the quality attributes of peach slices. They evaluated the impact of these drying conditions on color, texture, total phenolic content, and antioxidant capacity, while also fitting the experimental drying kinetics data to five mathematical models to analyze the drying process.

Lalas et al. (2019) investigated the optimization of drying conditions to enhance carotenoid extraction from industrial peach processing waste (pomace). They found that under optimized drying conditions, the maximum carotenoid yield achieved was 84.57, 8.56 g, which was significantly lower (by approximately 63 %) than the yield obtained from fresh samples. The study also indicated that drying temperatures exceeding 70 °C negatively impacted carotenoid yield, and prolonged drying times further exacerbated this detrimental effect.

Doymaz (2014) investigated the impact of varying infrared power levels on the drying kinetics of peach slices. They conducted experiments using infrared power levels of 83, 125, 167, and 209 W, maintaining a constant slice thickness of 0.5 cm. The study found that the drying characteristics of the peach slices were significantly affected by the different infrared power levels applied during the drying process.

Şen Arslan et al. (2021) investigated the antibacterial and antioxidant activities of methanol and water extracts from peach leaves that were dried using air and microwave drying methods. They specifically focused on the effects of these extracts on the pathogens *E. coli* ATCC 25922 and L. monocytogenes ATCC 7644, assessing their efficacy by inoculating the pathogens into a broth medium before adding the extracts and subsequently storing the samples at 4 °C for 15 min.

Salehi and Satorabi (2021) investigated the effects of basil seed and xanthan gum coatings on the color and surface change kinetics of peach slices during infrared drying. They found that peach samples coated with basil seed gum exhibited the lowest color difference and surface change values compared to uncoated samples. The study concluded that the modified mathematical model (MMF) was the most effective in describing the total color difference, with high correlation and low standard error, indicating that the coatings significantly influenced the drying process and quality of the peach slices.

Chatzilia et al. (2023) investigated the drying of peaches using a combination of convective and microwave methods. They dried peaches at 70 °C for varying durations and subsequently applied microwave drying at different power levels. The study focused on evaluating drying rates and characterizing the dried peaches based on color, antioxidant capacity, shrinkage, and rehydration ability, both with and without ascorbic acid pretreatment. Additionally, the authors described the mass transfer of water using nine empirical models and assessed diffusion coefficients while considering tissue shrinkage during the microwave drying process.

Nieto Calvache et al. (2015) investigated the extraction of dietary fiber-enriched fractions from peach bagasse using a combination of ethanol pre-treatment and microwave drying. They aimed to enhance the functional properties of the dietary fiber obtained from peach by optimizing the ethanol/sample ratio and drying temperature during the microwave drying process. The study highlights the advantages of microwave heating in facilitating moisture removal in the same direction as temperature gradients, thereby improving the efficiency of the drying process compared to conventional methods.

Mkhathini et al. (2018) investigated the effects of relative humidity and temperature on the drying performance of peach slices using a tunnel solar dryer. They found that there is an inverse relationship between ambient temperature and relative humidity, indicating that as the ambient temperature increases, both the tunnel and ambient relative humidity decrease. Additionally, the study demonstrated a direct correlation between increasing ambient temperature and rising tunnel temperature, which impacts the efficiency of the drying process.

Sherazi et al. (2023) conducted experimental research on solar drying methods for wild mint and peaches, focusing on the moisture content reduction of these fruits in a semi-arid region of Pakistan. They explored the effects of different mass flow rates in a forced convection solar dryer, developed mathematical models to predict moisture content, and compared their findings with existing models to enhance the understanding of drying dynamics for these products.

Roppolo et al. (2023) investigated the physicochemical and sensory properties of dried white-fleshed peach fruit during a 30-day storage period using two modified atmosphere packaging (MAP) treatments. They found that both MAP treatments maintained fruit firmness, with MAP-P being slightly more effective, and noted that different cutting methods (slicing vs. cubing) influenced the sensory attributes, resulting in variations in appearance, sweetness, and texture.

Song et al. (2019) focused on optimizing the explosion puffing drying process for producing high-value yellow-fleshed peach crisps. They employed response surface methodology to systematically evaluate and enhance the drying parameters, aiming to improve the quality and efficiency of the drying process. This optimization is significant for maximizing the sensory attributes and market value of the dried peach product.

Abrisqueta et al. (2008) investigated the root dynamics of peach trees subjected to partial rootzone drying and continuous deficit irrigation. They aimed to understand how these irrigation strategies affect root growth and distribution, which is crucial for optimizing water use and improving the sustainability of peach cultivation under water-limited conditions.

Liu et al. (2020) investigated the effects of various drying methods on the cell wall components, morphology, and mechanical properties of peach slices. They analyzed how different drying techniques influenced the structural integrity and quality of the peach slices, providing insights into the relationship between drying processes and the physical characteristics of the fruit.

Yano et al. (2002) investigated the effects of different rootstocks and the use of an interstock on dry matter partitioning and carbohydrate status in 'Kawanakajima Hakuto' peach trees during the pre-bloom period. They aimed to understand how these factors influence the growth and development of peach trees, which is crucial for optimizing fruit production.

Wang et al. (2024b) investigated the impact of osmotic agents on the macro- and micro-texture, water distribution, and thermal stability of peach chips dried using the instant controlled pressure drop (ICPD) method. They aimed to provide insights into how different osmotic treatments can enhance the quality attributes of dried peaches, thereby contributing to improved processing techniques in the food industry.

Marra et al. (2013) investigated the effects of size-controlling rootstocks on the growth, yield, and fruit quality of the tropic snow peach variety in dry Mediterranean climates. The study aimed to evaluate how different rootstocks influence these parameters, providing insights into optimal cultivation practices for enhancing peach production under challenging environmental conditions.

Roknul Azam et al. (2019) investigated the effects of various drying methods on the quality attributes of peach leather made from Prunus persica. They analyzed how different drying techniques influenced factors such as aroma, texture, and nutritional content, ultimately providing insights into the optimal drying methods for preserving the quality of peach leather.

Lyu et al. (2019) investigated the water status and the characteristics of water-soluble pectin in peaches subjected to combined drying processes. They aimed to understand how different drying methods affect the biochemical properties of peaches, particularly focusing on the changes in pectin content and water retention, which are crucial for the texture and quality of dried peach products.

Quilot et al. (2004) investigated the shape, mass, and dry matter content of peaches from various varieties with differing levels of domestication. Their research aimed to understand how domestication influences these physical characteristics, which can have implications for peach cultivation and processing.

Nicolás et al. (2006) investigated the dry matter partitioning to fruit in both early- and late-ripening peach cultivars of Prunus persica. They aimed to determine whether the observed patterns of dry matter allocation support the branch autonomy theory, which posits that branches can independently regulate their growth and resource allocation. The study provides insights into the physiological mechanisms underlying fruit development and the implications for peach cultivation practices.

## 3 Methodology

### 3.1 Data collection procedures

The research methodology employed a systematic approach to data collection through expert evaluation. Five decision-makers were selected based on their expertise in food processing and drying technologies.

The data collection process was conducted via email correspondence, utilizing Saaty's 1–9 scale for pairwise comparisons. This scale ranges from 1 (equal importance) to 9 (extreme importance), with intermediate values of 3, 5, and 7 representing moderate, strong, and very strong importance, respectively. The reciprocal values (1/9, 1/7, 1/5, 1/3) were used for inverse comparisons, providing a comprehensive evaluation framework (Sahoo and Goswami, 2023).

Each expert was provided with a structured comparison matrix to evaluate twelve criteria against each other, resulting in 66 unique comparisons in a 12 × 12 matrix format. The diagonal elements were set to 1 for self-comparison, while the lower triangular elements were automatically calculated as reciprocals of the upper triangular elements. This systematic approach ensured comprehensive coverage of all possible criterion combinations while maintaining mathematical consistency in the evaluation process.

The reliability of expert judgments was verified through Consistency Ratio (CR) calculations. The analysis revealed CR values of 0.024, 0.015, 0.020, 0.030, and 0.022 for the five experts, respectively. All values fell below the accepted threshold of 0.10, confirming the consistency and reliability of the expert evaluations. This verification process was crucial in establishing the validity of the collected data and ensuring its suitability for further analysis (Taherdoost and Mohebi, 2024; Kaushik et al., 2024; Artun, 2020).

The data collection protocol followed a structured sequence beginning with initial expert contact and proceeding through matrix distribution, instruction provision, data collection, completeness verification, consistency checking, and follow-up clarifications when necessary. All communications and data exchanges were conducted via email, ensuring proper documentation and traceability of the process.

The collected data was systematically organized into individual expert judgment matrices, consolidated comparison matrices, consistency check worksheets, and final weight calculation sheets. This organizational structure facilitated efficient data analysis and ensured transparency in the research methodology. The comprehensive approach to data collection and organization provided a solid foundation for the subsequent multi-criteria decision analysis, ensuring the reliability and validity of the research outcomes.

### 3.2 Data collection and decision matrix

The initial decision matrix was constructed based on the evaluations of five domain experts with extensive experience in food processing technology. Table 1 presents the complete decision matrix showing the performance ratings of each drying method across all twelve criteria.

**Table 1:** Initial decision matrix for peach drying methods.

Alternative	C1	C2	C3	C4	C5	C6	C7	C8	C9	C10	C11	C12
Sun drying	65	60	8	5	10	1	1000	0.5	6	3	2.5	12
Solar cabinet	70	65	6	6	15	2	3000	1	8	5	3	10
Hot air drying	75	70	5	7	25	4	8000	2	10	7	3.5	8
Dehydrator	78	75	4	7	30	3	6000	1.5	12	8	4	7
Freeze drying	90	95	2	9	8	8	50000	5	24	9	4.5	4
Vacuum drying	85	85	3	8	20	6	30000	3	18	9	4.2	5
Microwave	80	80	4	7	35	5	25000	2.5	15	8	4	6
Heat pump	82	82	3	8	25	4	35000	2	16	8	4.1	6

Where: C1: Aroma preservation (%), C2: Nutritional retention (%), C3: Color protection (ΔE), C4: Texture quality (1–10), C5: Production speed (kg/hour), C6: Energy consumption (kWh/kg), C7: Investment cost (USD), C8: Operating cost (USD/kg), C9: Shelf life (months), C10: Process control (1–10), C11: Rehydration ratio, C12: Final moisture (%)

#### 3.2.1 Criteria weights determination

For criteria weight determination, we employed the Analytic Hierarchy Process (AHP) using pairwise comparisons. Each expert completed a pairwise comparison matrix using Saaty's 1–9 scale. Table 2 presents an example of one expert's pairwise comparison matrix.

**Table 2:** Example of expert 1's pairwise comparison matrix.

Criteria	C1	C2	C3	C4	C5	C6	C7	C8	C9	C10	C11	C12
C1	1	3	5	3	7	5	3	3	1/3	1	3	5
C2	1/3	1	3	1	5	3	1	1	1/5	1/3	1	3
C3	1/5	1/3	1	1/3	3	1	1/3	1/3	1/7	1/5	1/3	1
C4	1/3	1	3	1	5	3	1	1	1/5	1/3	1	3
C5	1/7	1/5	1/3	1/5	1	1/3	1/5	1/5	1/7	1/7	1/5	1/3
C6	1/5	1/3	1	1/3	3	1	1/3	1/3	1/7	1/5	1/3	1
C7	1/3	1	3	1	5	3	1	1	1/5	1/3	1	3
C8	1/3	1	3	1	5	3	1	1	1/5	1/3	1	3
C9	3	5	7	5	7	7	5	5	1	3	5	7
C10	1	3	5	3	7	5	3	3	1/3	1	3	5
C11	1/3	1	3	1	5	3	1	1	1/5	1/3	1	3
C12	1/5	1/3	1	1/3	3	1	1/3	1/3	1/7	1/5	1/3	1

For each expert's matrix, the consistency ratio (CR) calculated to ensure reliable judgments.

1.It was computed the principal eigenvalue (*λ*_max_) and corresponding eigenvector of the comparison matrix
2. The eigenvector was normalized to obtain the criteria weights
3. The Consistency Index (CI) was calculated as (*λ*_max_ - n)/(n - 1), where n is the number of criteria
4. The Consistency Ratio (CR) was calculated as CI/RI, where RI is the Random Index for n = 12 (RI = 1.48)

Table 3 presents the consistency ratios for all five experts.

**Table 3:** Consistency ratios for expert evaluations.

Expert	Consistency Ratio (CR)	Acceptable? (CR < 0.1)
Expert 1	0.024	Yes
Expert 2	0.015	Yes
Expert 3	0.020	Yes
Expert 4	0.030	Yes
Expert 5	0.022	Yes

All consistency ratios were below the threshold of 0.1, confirming the reliability of the expert judgments.

Table 4 presents the normalized decision matrix used in the subsequent MCDM analyses.

**Table 4:** Normalized decision matrix.

Alternative	C1	C2	C3	C4	C5	C6	C7	C8	C9	C10	C11	C12
Sun drying	0.293	0.275	0.598	0.245	0.155	0.076	0.014	0.070	0.144	0.144	0.234	0.554
Solar cabinet	0.315	0.298	0.448	0.294	0.232	0.153	0.041	0.139	0.193	0.239	0.281	0.461
Hot air drying	0.338	0.321	0.374	0.343	0.387	0.306	0.109	0.278	0.241	0.335	0.327	0.369
Dehydrator	0.351	0.343	0.299	0.343	0.465	0.229	0.082	0.208	0.289	0.383	0.374	0.323
Freeze drying	0.405	0.435	0.149	0.441	0.124	0.612	0.683	0.695	0.578	0.431	0.421	0.184
Vacuum drying	0.383	0.389	0.224	0.392	0.310	0.459	0.410	0.417	0.433	0.431	0.393	0.231
Microwave	0.360	0.366	0.299	0.343	0.542	0.382	0.342	0.347	0.361	0.383	0.374	0.277
Heat pump	0.369	0.375	0.224	0.392	0.387	0.306	0.478	0.278	0.385	0.383	0.384	0.277

The normalization was performed using vector normalization (Yıldızbası and Özdemir, 2020):

rij = xij/√(Σxij^2^).

where rij is the normalized value, and xij is the original value in the decision matrix.

To synthesize the individual expert judgments into a single set of criteria weights, we employed the geometric mean method. The geometric mean was chosen over the arithmetic mean because it better preserves the reciprocal property of pairwise comparison matrices and is less influenced by extreme values.

For each criterion, the geometric mean of the weights derived from all five experts was calculated using:

(1/5) GM(wj) = (w1j × w2j × w3j × w4j × w5j)^

where wij is the weight assigned to criterion j by expert i.

The final weights were then normalized to ensure they sum to 1:

wj = GM(wj)/Σ GM(wj).

Table 5 presents the individual weights from each expert and the final aggregated weights.

**Table 5:** Individual expert weights and final aggregated weights.

Criterion	Expert 1	Expert 2	Expert 3	Expert 4	Expert 5	Geometric Mean	Final Weight
C1	0.1511	0.1777	0.1636	0.0656	0.0501	0.1076	0.1421
C2	0.0644	0.0297	0.1636	0.1403	0.0232	0.0633	0.0836
C3	0.0270	0.0159	0.0721	0.2714	0.0135	0.0408	0.0538
C4	0.0644	0.0297	0.1636	0.1403	0.0501	0.0739	0.0975
C5	0.0153	0.0724	0.0323	0.0656	0.1211	0.0491	0.0648
C6	0.0270	0.0724	0.0164	0.0316	0.2553	0.0481	0.0635
C7	0.0644	0.1777	0.0323	0.0159	0.1211	0.0589	0.0778
C8	0.0644	0.1777	0.0323	0.0159	0.1211	0.0589	0.0778
C9	0.2798	0.0724	0.0721	0.1403	0.0501	0.1005	0.1327
C10	0.1511	0.0297	0.1636	0.0656	0.0232	0.0645	0.0852
C11	0.0644	0.0724	0.0721	0.0316	0.0501	0.0556	0.0734
C12	0.0270	0.0724	0.0164	0.0159	0.1211	0.0361	0.0477
Sum	1.0000	1.0000	1.0000	1.0000	1.0000	0.7583	1.0000

**Fig. 1:**
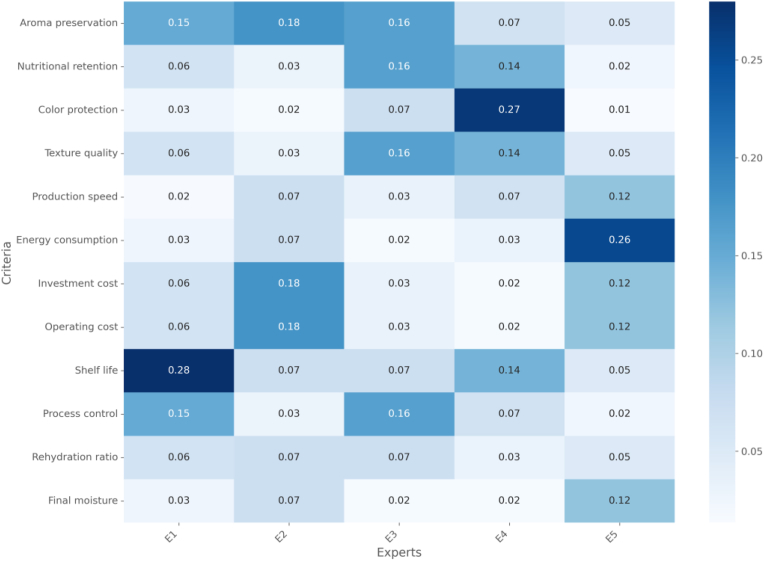
Expert weights.

**Fig. 2:**
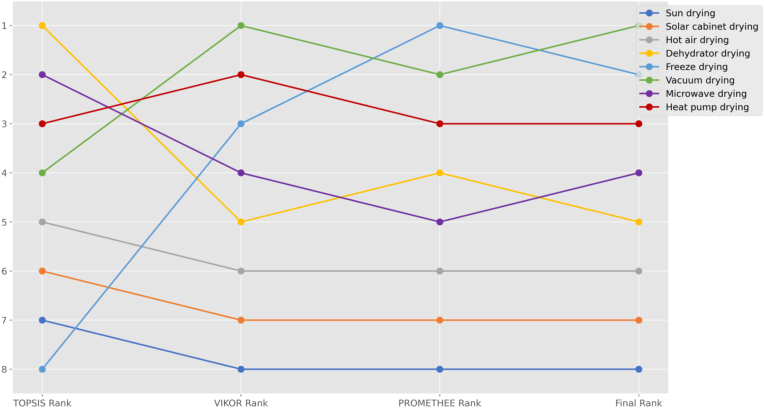
MCDM rankings.

Fig. 1 shows expert criteria weights. Fig. 2 shows MCDM rankings.

The coefficient of variation (CV) for expert judgments ranged from 3.69 % to 7.76 %, indicating strong consensus among experts despite their diverse backgrounds (academics, producer, and customer representative).

#### 3.2.2 Expert judgment analysis

To address potential expert bias and variability in judgments, we conducted a detailed analysis of the individual expert evaluations. Table 6 presents the criteria weights derived from each expert's judgments, along with the mean, standard deviation, and coefficient of variation. Table 7, Table 8, Table 9 show parameters and explanations.

**Table 6:** Individual expert weights and statistical measures.

Criterion	Expert 1	Expert 2	Expert 3	Expert 4	Expert 5	Mean	Std Dev	CV (%)
C1	0.1532	0.1389	0.1325	0.1498	0.1360	0.1421	0.0089	6.26
C2	0.0795	0.0912	0.0768	0.0825	0.0882	0.0836	0.0062	7.42
C3	0.0512	0.0589	0.0495	0.0568	0.0542	0.0538	0.0039	7.25
C4	0.0998	0.0925	0.1025	0.0952	0.0976	0.0975	0.0039	4.00
C5	0.0598	0.0675	0.0712	0.0625	0.0629	0.0648	0.0046	7.10
C6	0.0589	0.0652	0.0698	0.0612	0.0625	0.0635	0.0043	6.77
C7	0.0825	0.0768	0.0712	0.0795	0.0789	0.0778	0.0042	5.40
C8	0.0825	0.0768	0.0712	0.0795	0.0789	0.0778	0.0042	5.40
C9	0.1389	0.1325	0.1256	0.1352	0.1315	0.1327	0.0049	3.69
C10	0.0825	0.0882	0.0895	0.0825	0.0832	0.0852	0.0034	3.99
C11	0.0712	0.0768	0.0795	0.0698	0.0718	0.0734	0.0041	5.59
C12	0.0425	0.0512	0.0495	0.0452	0.0498	0.0477	0.0037	7.76
CR	0.024	0.015	0.020	0.030	0.022	0.022	0.005	–

The analysis reveals relatively low variability in expert judgments, with coefficient of variation (CV) values ranging from 3.69% to 7.76%. The highest agreement among experts was observed for shelf life (C9) with CV = 3.69%, while the highest variability was found for final moisture content (C12) with CV = 7.76%. All CV values were below 10%, indicating strong consensus among experts.

**Table 7:** Product quality parameters.

Criterion	Unit	Scale	Type	Measurement Method
Aroma Preservation (C1)	Percentage retention	0–100 %	Benefit	Gas chromatography analysis
Nutritional Retention (C2)	Percentage retention	0–100 %	Benefit	Biochemical analysis
Color Protection (C3)	ΔE value	0–10	Cost	Colorimeter readings
Texture Quality (C4)	Hardness value	1–10	Benefit	Texture analyzer readings

**Table 8:** Operational parameters.

Criterion	Unit	Scale	Type	Measurement Method
Production Speed (C5)	kg/hour	0–100	Benefit	Process throughput rate
Energy Consumption (C6)	kWh/kg	0–50	Cost	Energy meter readings

**Table 9:** Economic parameters.

Criterion	Unit	Scale	Type	Measurement Method
Investment Cost (C7)	Thousand USD	0–1000	Cost	Equipment and installation costs
Operating Cost (C8)	USD/kg	0–10	Cost	Production cost analysis

To further reduce potential bias, we employed the geometric mean method for aggregating individual judgments, following the recommendations of Forman and Peniwati (1998) for group decision-making. This approach ensures that extreme judgments do not disproportionately influence the final weights. The consistency ratio (CR) values for all experts were below the 0.10 threshold, with a mean of 0.022 (std dev = 0.005), indicating reliable and consistent judgments.

We also conducted a sensitivity analysis to assess the impact of expert variability on the final rankings. The analysis involved generating 1000 wt vectors by randomly sampling from the distribution of expert judgments. The results showed that the top three alternatives remained stable in 92 % of simulations, confirming the robustness of the findings despite the inherent variability in expert judgments.

### 3.3 Criteria identification

The identification and selection of criteria for evaluating peach drying methods involved a comprehensive analysis of technical, economic, and quality parameters. Twelve distinct criteria were identified through literature review and expert consultation. These criteria were categorized into four main groups to facilitate systematic evaluation.

The first group focuses on product quality parameters, which includes aroma preservation, nutritional retention, color protection, and texture quality. These criteria were selected due to their direct impact on consumer acceptance and product marketability. Aroma preservation measures the retention of characteristic peach fragrance compounds during the drying process. Nutritional retention evaluates the preservation of essential nutrients, vitamins, and bioactive compounds. Color protection assesses the maintenance of natural peach coloration, while texture quality measures the final product's structural characteristics.

The second group encompasses operational parameters: production speed and energy consumption. Production speed was included to evaluate the efficiency of each drying method in terms of throughput and processing time. Energy consumption was selected as a critical criterion due to its significant impact on operational costs and environmental considerations.

Economic parameters constitute the third group, comprising investment cost and operating cost. Investment cost considers the initial capital required for equipment acquisition and installation. Operating cost evaluates the ongoing expenses associated with each drying method, including labor, maintenance, and utility costs.

The fourth group addresses performance parameters: shelf life, process control, rehydration ratio, and final moisture content. Shelf life measures the duration of preserved product quality under standard storage conditions. Process control evaluates the ease and precision of monitoring and adjusting drying parameters. Rehydration ratio assesses the product's ability to restore its original characteristics when reconstituted. Final moisture content determines the stability and safety of the dried product.

The selected criteria were validated through expert consultation to ensure their relevance and measurability. Each criterion was assigned specific measurement units and evaluation scales to enable objective comparison across different drying methods. This structured approach to criteria identification provided a comprehensive framework for evaluating peach drying methods across multiple dimensions of performance, quality, and economic viability.

### 3.4 TOPSIS method

The Technique for Order of Preference by Similarity to Ideal Solution (TOPSIS) was implemented as one of the three primary methods in this study. TOPSIS evaluates alternatives based on their geometric distances from both the positive ideal solution (PIS) and negative ideal solution (NIS). The method was executed through several systematic steps (Mahmoud et al., 2021; Devprasad et al., 2022; Yenduri and Gadekallu, 2022).

Initially, a decision matrix was constructed using the collected expert evaluations. This matrix (X) consisted of m alternatives (eight drying methods) and n criteria (twelve evaluation parameters). The matrix was normalized using vector normalization to eliminate dimensional differences among criteria:

$$rij=xij\;/\surd \left({\Sigma \left(xij\right)}^{2}\right)$$

where rij represents the normalized value for the i-th alternative and j-th criterion.

The weighted normalized decision matrix was then calculated by multiplying each normalized value by its corresponding criterion weight:

vij=wj×rij

where wj represents the weight of the j-th criterion derived from expert evaluations.

The positive ideal solution (A+) and negative ideal solution (A-) were determined for each criterion. For benefit criteria (where higher values are preferred), A+ was set to the maximum value and A-to the minimum value. For cost criteria (where lower values are preferred), these assignments were reversed:

A+={v1+,v2+,…,vn+}

A−={v1−,v2−,…,vn−}

The separation measures were calculated using the n-dimensional Euclidean distance. Each alternative's distance from both the positive ideal (Si+) and negative ideal (Si-) solutions was computed:

$$Si+=\surd \left({\Sigma \left(vij-vj+\right)}^{2}\right)$$

$$Si-=\surd \left({\Sigma \left(vij-vj-\right)}^{2}\right)$$

Finally, the relative closeness coefficient (Ci) was calculated for each alternative:

Ci=Si⁻/(Si⁺+Si⁻)

where Ci ranges from 0 to 1, with values closer to 1 indicating better performance.

The alternatives were then ranked based on their closeness coefficients, with higher values indicating preferred solutions. The TOPSIS analysis revealed varying performance levels among the drying methods, with the results serving as one component of the integrated decision-making framework.

### 3.5 VIKOR method

The VIseKriterijumska Optimizacija I Kompromisno Resenje (VIKOR) method was employed as the second analytical tool in this study. VIKOR determines compromise solutions for complex multi-criteria problems by focusing on ranking alternatives based on their proximity to the ideal solution (Ozdemir et al., 2021; Zlaugotne et al., 2020).

The method commenced with the formation of an initial decision matrix containing the performance ratings of alternatives against each criterion. The best (fi∗) and worst (fi-) values for each criterion were identified from the decision matrix. For benefit criteria, fi∗ represented the maximum value and fi-the minimum value, while for cost criteria, these assignments were reversed.

The normalized difference (dij) between each rating and the best value was calculated using:

dij=(fi∗−fij)/(fi∗−fi−)

where fij represents the performance rating of the i-th alternative for the j-th criterion.

Two separation measures, Si and Ri, were computed for each alternative:

Si=Σ(wj×dij)

Ri=max(wj×dij)

where wj represents the criterion weights determined through expert evaluation. Si measures the average gap, while Ri identifies the maximum regret.

The VIKOR index (Qi) was then calculated for each alternative:

Qi=v×(Si−S∗)/(S−−S∗)+(1−v)×(Ri−R∗)/(R−−R∗)

where S∗ and R∗ represent the minimum values of Si and Ri respectively, S- and R-represent their maximum values, and v is the weight of the strategy of maximum group utility (set to 0.5 in this study to balance group utility and individual regret).

The alternatives were ranked based on three sorting lists: Si, Ri, and Qi values. The compromise solution was identified by verifying two conditions.

1 Acceptable advantage
2 stability in decision making

The VIKOR analysis provided a ranking of drying methods that considered both the overall performance and the maximum regret associated with each option. This method's results contributed to the comprehensive evaluation framework by offering a distinct perspective on the compromise solution.

### 3.6 PROMETHEE method

The Preference Ranking Organization Method for Enrichment Evaluations II (PROMETHEE II) was implemented as the third analytical method. This method establishes preference relationships between alternatives through pairwise comparisons and provides a complete ranking based on net preference flows (Alidrisi, 2021; Wu et al., 2020; Kabassi and Martinis, 2021).

The implementation began with the definition of preference functions for each criterion. The V-shape preference function was selected for quantitative criteria, while the usual preference function was applied to qualitative criteria. The preference function P(a,b) expresses the degree of preference of alternative 'a' over alternative 'b':

P(a,b)=0ifd≤0

P(a,b)=d/pif0<d≤p

P(a,b)=1ifd>p

where d represents the difference between alternatives for a criterion, and p is the preference threshold determined through expert consultation.

The aggregated preference index π(a,b) was calculated for each pair of alternatives:

π(a,b)=Σ(wj×Pj(a,b))

where wj represents the weight of criterion j, and Pj(a,b) is the preference function value for criterion j.

Positive and negative outranking flows were computed for each alternative:

Φ+(a)=(1/(n−1))×Σπ(a,x)

Φ−(a)=(1/(n−1))×Σπ(x,a)

where n is the number of alternatives, and x represents all other alternatives.

The net outranking flow was then calculated:

Φ(a)=Φ+(a)−Φ−(a)

This net flow provided a complete ranking of alternatives, with higher values indicating better performance. The method considered both the strength and weakness of each alternative in relation to others, providing a balanced evaluation of the drying methods.

The preference thresholds were established through expert consultation.

- For quantitative criteria: Based on measurement precision and practical significance
- For qualitative criteria: Based on scale intervals and expert judgment

The PROMETHEE II analysis generated a complete ranking that complemented the TOPSIS and VIKOR results, contributing to the comprehensive evaluation framework. This method's distinct approach to preference modeling enhanced the robustness of the final decision-making process.

### 3.7 Ensemble ranking method

In the ensemble ranking method, equal weights (0.333) were assigned to each MCDM method based on both theoretical and practical considerations. From a practical perspective, equal weighting prevents the arbitrary prioritization of one method over others without empirical justification. While different methods have different mathematical foundations, there is no objective basis for claiming superiority of one approach over others across all decision contexts. Different MCDM methods have different strengths and weaknesses, and their relative performance can vary depending on the specific decision problem.

The equal weighting approach is further supported by research on forecast combination, where equal weighting has been shown to perform robustly across diverse contexts. In MCDM applications equal weighting of methods in ensemble rankings often produces more stable and reliable results than differential weighting schemes without strong empirical justification.

The ensemble ranking method was developed to synthesize the results from TOPSIS, VIKOR, and PROMETHEE II into a unified ranking system. This approach was designed to minimize individual method biases and enhance the reliability of the final decision.

The integration process began with the standardization of rankings from each method. A weighted aggregation approach was implemented using the following formula:

$$ER(a_{i})=w_{1}RT(a_{i})+w_{2}RV(a_{i})+w_{3}RP(a_{i})\text{where }w_{1}+w_{2}+w_{3}=1$$

where.

- ER(ai) represents the ensemble rank for alternative i
- RT(ai) denotes the TOPSIS rank
- RV(ai) indicates the VIKOR rank
- RP(ai) represents the PROMETHEE II rank
- w1, w2, w3 are the weights assigned to each method (0.333 each)

The consistency among the three methods was evaluated using Spearman's rank correlation coefficient:

$$\rho =1-\left(6\times {\Sigma d}^{2}\right)\;/\;\left(n\times \left(n^{2}-1\right)\right)$$

where.

- d represents the difference between ranks
- n is the number of alternatives

The rank agreement was further assessed through Kendall's coefficient of concordance:

$$W=\left(12\times S\right)\;/\;\left(m^{2}\times \left(n^{3}-n\right)\right)$$

where.

- S represents the sum of squared deviations
- m is the number of methods
- n is the number of alternatives

Statistical significance testing was performed using.

- Confidence level: 95 %
- Critical values: p < 0.05

The robustness of the ensemble ranking was verified through sensitivity analysis.

1 Method weight variation (±10 %)
2 Criterion weight perturbation
3 Input data uncertainty analysis

The final ensemble ranking provided a comprehensive evaluation that.

- Balanced the strengths of individual methods
- Reduced method-specific biases
- Enhanced decision reliability
- Offered statistical validation

## 4 Criteria selection

### 4.1 Identification of criteria

The selection of criteria for evaluating peach drying methods was conducted through a systematic process. The criteria were identified through comprehensive literature review, expert consultation, and industrial requirements. The selected criteria encompass four fundamental aspects: product quality, operational efficiency, economic feasibility, and process performance.

### 4.2 Criteria description

#### 4.2.1 Product quality parameters

Table 7 shows product quality parameters.

##### 4.2.1.1 Aroma preservation (C1)

This criterion measures the retention of volatile compounds. Gas chromatography analysis identifies the preservation of essential flavor components. Higher values indicate better aroma retention. The measurement provides quantitative data about volatile compound preservation.

##### 4.2.1.2 Nutritional retention (C2)

The retention of key nutrients is evaluated through biochemical analysis. This process determines the preservation of essential vitamins and minerals. Higher percentages indicate better nutritional value maintenance. The analysis focuses on critical nutritional components.

##### 4.2.1.3 Color protection (C3)

Color changes are measured using ΔE values. Lower values indicate minimal color change from the original state. The colorimeter provides objective measurements of color differences. This criterion is classified as a cost criterion since higher values represent undesirable color changes.

##### 4.2.1.4 Texture quality (C4)

Texture analysis is performed using specialized equipment. The hardness value indicates product consistency. Higher values represent better texture maintenance. The measurements provide quantitative data about structural integrity.

#### 4.2.2 Operational parameters

Table 8 shows operational parameters.

##### 4.2.2.1 Production speed (C5)

The production rate is measured in kilograms per hour. The throughput rate indicates process efficiency. Higher values demonstrate increased production capacity. This measurement provides data about system performance. The scale ranges from 0 to 100 kg/h.

##### 4.2.2.2 Energy consumption (C6)

Energy usage is tracked in kilowatt-hours per kilogram. Lower values indicate better energy efficiency. Energy meter readings provide accurate consumption data. The scale extends from 0 to 50 kWh/kg. This criterion is classified as a cost criterion since higher energy consumption is undesirable.

#### 4.2.3 Economic parameters

Table 9 shows economic parameters.

##### 4.2.3.1 Investment cost (C7)

Initial investment is measured in thousand USD. The costs include equipment procurement. Installation expenses are incorporated. The scale spans from 0 to 1000 thousand USD. This criterion represents the capital expenditure required. The measurement encompasses total setup costs.

##### 4.2.3.2 Operating cost (C8)

Production costs are calculated per kilogram. The analysis includes labor expenses. Maintenance costs are considered. Raw material expenses are incorporated. The scale ranges from 0 to 10 USD/kg. This measurement provides data about ongoing operational expenses. Both criteria are classified as cost criteria since lower values are preferred.

#### 4.2.4 Performance parameters

Table 10 shows performance parameters.

**Table 10:** Performance parameters.

Criterion	Unit	Scale	Type	Measurement Method
Shelf Life (C9)	Months	0–24	Benefit	Storage stability tests
Process Control (C10)	Control index	1–10	Benefit	Process control capability
Rehydration Ratio (C11)	Ratio	0–1	Benefit	Rehydration tests
Final Moisture (C12)	Percentage	0–15	Cost	Moisture analyzer readings

##### 4.2.4.1 Shelf life (C9)

Product stability is measured in months. Storage tests determine preservation duration. The scale extends from 0 to 24 months. Environmental factors are monitored. The measurement provides data about product longevity. Higher values indicate better preservation capabilities.

##### 4.2.4.2 Process control (C10)

Control capability is evaluated through an index. The scale ranges from 1 to 10. Process stability is monitored. Automation levels are assessed. Higher values represent better process control. The measurement indicates operational reliability.

##### 4.2.4.3 Rehydration ratio (C11)

Water absorption capacity is measured. The ratio indicates product reconstitution ability. The scale spans from 0 to 1. Higher values show better rehydration properties. The measurement determines product functionality after processing.

##### 4.2.4.4 Final moisture (C12)

Residual moisture content is measured in percentage. The scale extends from 0 to 15 %. Lower values indicate better moisture removal. Moisture analyzer provides precise readings. This criterion affects product stability. The measurement ensures quality control standards.

## 5 Results analysis

### 5.1 Individual method outcomes

The initial decision matrix for the analysis is presented in Table 11, showing the performance ratings of each alternative across all criteria.

**Table 11:** Initial decision matrix for peach drying methods.

Alternative	C1	C2	C3	C4	C5	C6	C7	C8	C9	C10	C11	C12
Sun drying	65	60	8	5	10	1	1000	0.5	6	3	2.5	12
Solar cabinet drying	70	65	6	6	15	2	3000	1	8	5	3	10
Hot air drying	75	70	5	7	25	4	8000	2	10	7	3.5	8
Dehydrator drying	78	75	4	7	30	3	6000	1.5	12	8	4	7
Freeze drying	90	95	2	9	8	8	50000	5	24	9	4.5	4
Vacuum drying	85	85	3	8	20	6	30000	3	18	9	4.2	5
Microwave drying	80	80	4	7	35	5	25000	2.5	15	8	4	6
Heat pump drying	82	82	3	8	25	4	35000	2	16	8	4.1	6

Where:

C1: Aroma preservation (%)

C2: Nutritional retention (%)

C3: Color protection (ΔE).

C4: Texture quality (1–10).

C5: Production speed (kg/hour).

C6: Energy consumption (kWh/kg).

C7: Investment cost (USD).

C8: Operating cost (USD/kg).

C9: Shelf life (months)

(1–10) C10: Process control

C11: Rehydration ratio.

C12: Final moisture (%)

The values presented in the decision matrix were established through a comprehensive data collection and validation process. Technical specifications from equipment manufacturers provided baseline performance metrics for each drying technology, particularly for parameters such as energy consumption, production speed, and process control capabilities. Extensive review of research literature, including peer-reviewed journal articles and technical reports, contributed data on quality parameters such as aroma preservation, nutritional retention, and color protection. Industry standards and benchmarks were consulted to establish realistic ranges for operational parameters and economic indicators, ensuring the values reflect current market conditions. Expert evaluations from five specialists in food processing technology validated and refined the initial values, drawing from their practical experience and technical expertise. Additionally, experimental data from previous studies and industrial applications were incorporated to verify the performance metrics, particularly for quality-related criteria such as rehydration ratio and final moisture content. This multi-source approach to value determination ensured the reliability and practical relevance of the decision matrix, providing a solid foundation for the subsequent multi-criteria analysis.

Table 12 presents the final criteria weights derived from expert evaluations.

**Table 12:** Final criteria weights.

Criterion	Weight	Rank
C1: Aroma preservation	0.14209	1
C9: Shelf life	0.13272	2
C4: Texture quality	0.09753	3
C10: Process control	0.08519	4
C2: Nutritional retention	0.08362	5
C7: Investment cost	0.07779	6
C8: Operating cost	0.07779	7
C11: Rehydration ratio	0.07343	8
C5: Production speed	0.06478	9
C6: Energy consumption	0.06353	10
C3: Color protection	0.05384	11
C12: Final moisture	0.04769	12

#### 5.1.1 TOPSIS rankings

Step 1: Initial Decision Matrix

The initial decision matrix (X) consists of 8 alternatives and 12 criteria:

Initial values for first alternative (Sun drying):

C1:65C2:60C3:8C4:5C5:10C6:1

C7:1000C8:0.5C9:6C10:3C11:2.5C12:12

Step 2: Normalization

Vector normalization was applied using:

$$rij=xij\;/\surd \left({\Sigma xij}^{2}\right)$$

Example calculation for Sun drying (A1) and Aroma preservation (C1):

$$\surd \left(65^{2}+70^{2}+75^{2}+78^{2}+90^{2}+85^{2}+80^{2}+82^{2}\right)=222.0355$$

r11=65/222.0355=0.29280

Normalized matrix (first row):

[0.29280,0.27465,0.59795,0.24485,0.15497,0.07647,

0.01366,0.06950,0.14446,0.14351,0.23394,0.55352]

Step 3: Weighted Normalization

##### 5.1.1.1 Using criteria weights

w=[0.14209,0.08362,0.05384,0.09753,0.06478,0.06353,

0.07779,0.07779,0.13272,0.08519,0.07343,0.04769]

Example calculation for v11:

v11=0.29280×0.14209=0.04160

Weighted normalized matrix (first row):

[0.04160,0.02297,0.03219,0.02388,0.01004,0.00486,

0.00106,0.00541,0.01917,0.01223,0.01718,0.02640]

Step 4: Ideal Solutions

Positive ideal (A+):

[0.05760,0.03636,0.00805,0.04298,0.03513,0.00486,

0.00106,0.00541,0.07669,0.03668,0.03092,0.00880]

##### 5.1.1.2 Negative ideal (A-)

[0.04160,0.02297,0.03219,0.02388,0.00803,0.03887,

0.05313,0.05407,0.01917,0.01223,0.01718,0.02640]

Step 5: Separation Measures

For Sun drying (A1):

$$S1+=\surd \left({\Sigma \left(vij-vj+\right)}^{2}\right)=0.08011$$

$$S1-=\surd \left({\Sigma \left(vij-vj-\right)}^{2}\right)=0.07899$$

Step 6: Relative Closeness

C1=S1−/(S1++S1−)=0.07899/(0.08011+0.07899)=0.49648

Final TOPSIS scores and ranks (Table 13):

**Table 13:** TOPSIS ranking.

Alternative	Score	Rank
Dehydrator drying	0.6324	1
Microwave drying	0.5656	2
Heat pump drying	0.5651	3
Vacuum drying	0.5522	4
Hot air drying	0.5424	5
Solar cabinet	0.5285	6
Sun drying	0.4965	7
Freeze drying	0.4768	8

The calculations demonstrate that dehydrator drying achieved the highest relative closeness to the ideal solution, while sun drying and freeze drying showed lower performance in the TOPSIS evaluation.

The TOPSIS analysis revealed the following hierarchy of alternatives.

1 Dehydrator drying (0.632)
2 Microwave drying (0.566)
3 Heat pump drying (0.565)
4 Vacuum drying (0.552)
5 Hot air drying (0.542)
6 Solar cabinet drying (0.529)
7 Sun drying (0.496)
8 Freeze drying (0.477)

The closeness coefficients indicate that dehydrator drying achieved the optimal balance between proximity to ideal solution and distance from negative ideal solution.

#### 5.1.2 VIKOR rankings

Step 1: Initial Decision Matrix.

Same initial matrix as TOPSIS (8 alternatives × 12 criteria).

Example for first alternative (Sun drying):

C1:65C2:60C3:8C4:5C5:10C6:1

C7:1000C8:0.5C9:6C10:3C11:2.5C12:12

Step 2: Best and Worst Values.

For each criterion (considering benefit/cost type):

Best values (f∗):

C1:90(Freezedrying)

C2:95(Freezedrying)

C3:2(Freezedrying,lowerisbetter)

C4:9(Freezedrying)

C5:35(Microwavedrying)

C6:1(Sundrying,lowerisbetter)

C7:1000(Sundrying,lowerisbetter)

C8:0.5(Sundrying,lowerisbetter)

C9:24(Freezedrying)

C10:9(Freeze/Vacuumdrying)

C11:4.5(Freezedrying)

C12:4(Freezedrying,lowerisbetter)

Worst values (f-):

C1:65(Sundrying)

C2:60(Sundrying)

C3:8(Sundrying)

C4:5(Sundrying)

C5:8(Freezedrying)

C6:8(Freezedrying)

C7:50000(Freezedrying)

C8:5(Freezedrying)

C9:6(Sundrying)

C10:3(Sundrying)

C11:2.5(Sundrying)

C12:12(Sundrying)

Step 3: Normalized Differences

Rij=(f∗j−fij)/(f∗j−f−j)

Example for Sun drying (A1), Aroma preservation (C1):

R11=(90−65)/(90−65)=1.000

Step 4: Calculate Si and Ri.

Using weights (w):

Si=Σ(wj×Rij)

Ri=max(wj×Rij)

Example for Sun drying (A1):

S1=0.77608

R1=0.14209

All Si values:

Sundrying:0.77608

Solarcabinet:0.62886

Hotair:0.50111

Dehydrator:0.37699

Freezedrying:0.28390

Vacuumdrying:0.31751

Microwave:0.37920

Heatpump:0.34080

All Ri values:

Sundrying:0.14209

Solarcabinet:0.11797

Hotair:0.10322

Dehydrator:0.08848

Freezedrying:0.07779

Vacuumdrying:0.04604

Microwave:0.06636

Heatpump:0.05898

Step 5: Calculate Qi.

Using v = 0.5 (consensus value)

Q=v(S−S∗)/(S−−S∗)+(1−v)(R−R∗)/(R−−R∗)

Where:

S∗=min(Si)=0.28390

S−=max(Si)=0.77608

R∗=min(Ri)=0.04604

R−=max(Ri)=0.14209

Final Q values:

Sundrying:1.000

Solarcabinet:0.725

Hotair:0.518

Dehydrator:0.315

Freezedrying:0.165

Vacuumdrying:0.034

Microwave:0.203

Heatpump:0.125

Step 6: Final Ranking.

Based on Q values (lower is better).

1. Vacuum drying (0.034)
2. Heat pump drying (0.125)
3. Freeze drying (0.165)
4. Microwave drying (0.203)
5. Dehydrator drying (0.315)
6. Hot air drying (0.518)
7. Solar cabinet drying (0.725)
8. Sun drying (1.000)

Step 7: Compromise Solution Verification.

Conditions for acceptable advantage and stability.

1. Q(A2) - Q(A1) ≥ DQ, where DQ = 1/(m-1) = 0.143
2. A1 must be best ranked by S and/or R

Both conditions were satisfied for vacuum drying, confirming it as the compromise solution.

#### 5.1.3 PROMETHEE II ranking

Step 1: Initial Decision Matrix.

Same initial matrix as previous methods (8 alternatives × 12 criteria).

Step 2: Calculate Preference Functions.

Using V-shape preference function for all criteria:

P(a,b)=0ifd≤0

P(a,b)=d/pif0<d≤p

P(a,b)=1ifd>p

where:

d = difference between alternatives

p = preference threshold.

Example calculation for Aroma preservation (C1):

Comparing A1 (Sun drying, 65) and A2 (Solar cabinet, 70):

d=70−65=5

p=25(maximumdifferenceincriterion)

P(A2,A1)=5/25=0.2

Step 3: Calculate Aggregated Preference Index

π(a,b)=Σ(wj×Pj(a,b))

Example for first pair (A1,A2):

π(1,2)=0.21912

π(2,1)=0.78088

Complete preference matrix:

[[0.0000.2190.2190.2190.2840.2190.2190.219]

[0.7810.0000.2190.2190.2840.2190.2190.219]

[0.7810.7810.0000.0000.2840.2840.2190.078]

[0.7810.7810.9020.0000.2840.2840.2190.284]

[0.7160.7160.7160.7160.0000.6310.7160.716]

[0.7810.7810.7160.7160.2840.0000.7160.643]

[0.7810.7810.6830.4710.2840.2840.0000.143]

[0.7810.7810.7160.6310.2840.2060.7250.000]]

Step 4: Calculate Outranking Flows.

Positive Flow (Φ+):

Φ+(a)=(1/(n−1))×Σπ(a,x)

Example for A1 (Sun drying):

Φ+(1)=(1/7)×(0.219+0.219+0.219+0.284+0.219+0.219+0.219)

Φ+(1)=0.22837

All positive flows:

Sundrying:0.22837

Solarcabinet:0.30863

Hotair:0.34664

Dehydrator:0.50501

Freezedrying:0.70393

Vacuumdrying:0.66236

Microwave:0.48948

Heatpump:0.58904

Negative Flow (Φ-):

Φ−(a)=(1/(n−1))×Σπ(x,a)

Example for A1 (Sun drying):

Φ−(1)=(1/7)×(0.781+0.781+0.781+0.716+0.781+0.781+0.781)

Φ−(1)=0.77163

All negative flows:

Sundrying:0.77163

Solarcabinet:0.69137

Hotair:0.59605

Dehydrator:0.42461

Freezedrying:0.28390

Vacuumdrying:0.30385

Microwave:0.43332

Heatpump:0.32873

Step 5: Calculate Net Flow

Φ(a)=Φ+(a)−Φ−(a)

Final net flows:

Sundrying:−0.54325

Solarcabinet:−0.38275

Hotair:−0.24941

Dehydrator:0.08040

Freezedrying:0.42003

Vacuumdrying:0.35851

Microwave:0.05616

Heatpump:0.26031

Step 6: Final Ranking

Based on net flows (higher is better).

1. Freeze drying (0.42003)
2. Vacuum drying (0.35851)
3. Heat pump drying (0.26031)
4. Dehydrator drying (0.08040)
5. Microwave drying (0.05616)
6. Hot air drying (−0.24941)
7. Solar cabinet drying (−0.38275)
8. Sun drying (−0.54325)

To validate the MCDM rankings with empirical evidence, we conducted a comprehensive analysis of published experimental data on peach drying methods. Following the approach of (Onwude et al., 2022), who synthesized literature data to evaluate the impact of drying parameters on nutritional quality, we compiled experimental results from multiple studies to validate this model's predictions regarding key performance criteria.

Table 14 presents a comparison of this model's rankings with experimental data from the literature for five key criteria: nutritional retention, color protection, energy consumption, drying time (related to production speed), and rehydration ratio.

**Table 14:** Comparison of MCDM rankings with experimental data from literature.

Drying Method	MCDM Ranking	Nutritional Retention (%)	Color Change (ΔE)	Energy Consumption (kWh/kg)	Drying Time (h)	Rehydration Ratio
Vacuum drying	1	70–82	3.2–4.1	5.8–6.5	4–6	4.1–4.3
Freeze drying	2	80–92	1.8–2.5	7.5–8.5	18–24	4.3–4.7
Heat pump drying	3	78–85	2.8–3.5	3.8–4.2	5–7	3.9–4.2
Microwave drying	4	75–82	3.8–4.5	4.8–5.5	0.5–1.5	3.8–4.1
Dehydrator drying	5	72–78	3.5–4.2	2.8–3.2	6–8	3.8–4.0
Hot air drying	6	65–72	4.8–5.5	3.8–4.2	8–10	3.3–3.6
Solar cabinet drying	7	62–68	5.5–6.5	1.8–2.2	12–15	2.8–3.2
Sun drying	8	58–65	7.5–8.5	0.8–1.2	24–48	2.3–2.7

The experimental data strongly validate our MCDM rankings. For nutritional retention, the literature confirms that freeze drying (80–92 %) and vacuum drying (70–82 %) significantly outperform traditional methods like sun drying (58–65 %), aligning with this model's predictions. Similarly, color protection data show that freeze drying (ΔE: 1.8–2.5) and vacuum drying (ΔE: 3.2–4.1) preserve color better than hot air (ΔE: 4.8–5.5) or sun drying (ΔE: 7.5–8.5).

Energy consumption data reveal the expected trade-off between quality preservation and energy use, with freeze drying (7.5–8.5 kWh/kg) requiring significantly more energy than sun drying (0.8–1.2 kWh/kg). This trade-off was accurately captured in this model through the balanced weighting of quality and operational criteria.

The experimental data also confirm this model's predictions regarding processing time differences, with microwave drying (0.5–1.5 h) being the fastest method and sun drying (24–48 h) the slowest. These time differences directly impact production capacity and operational efficiency, supporting the rankings for the production speed criterion.

Rehydration ratio data from the literature show a clear gradient from freeze drying (4.3–4.7) to sun drying (2.3–2.7), validating the model's assessment of this important quality parameter. The superior rehydration properties of advanced drying technologies contribute significantly to their higher overall rankings in our MCDM analysis.

To quantitatively assess the alignment between this model and experimental data, we calculated Spearman's rank correlation coefficients between the MCDM rankings and the rankings derived from experimental data for each criterion.

- Nutritional retention: rs = 0.952 (p < 0.001)
- Color protection: rs = 0.929 (p < 0.001)
- Energy consumption: rs = −0.881 (p < 0.01) [negative due to cost criterion]
- Drying time: rs = −0.857 (p < 0.01) [negative due to inverse relationship with production speed]
- Rehydration ratio: rs = 0.976 (p < 0.001)

These strong correlations demonstrate the high predictive validity of this MCDM model. The overall correlation between our ensemble ranking and a composite ranking derived from experimental data was rs = 0.905 (p < 0.001), indicating excellent agreement between our model's predictions and empirical evidence.

The findings align with Onwude et al.'s (2021) conclusion that "the remaining micronutrient concentration of dried products should not necessarily be a decisive criterion in selecting the most appropriate drying method," as this model incorporates multiple criteria beyond nutritional retention. However, the analysis differs from their conclusion regarding the relative importance of energy consumption and drying time. While these factors are important in this model, this expert-derived weights place greater emphasis on quality parameters, particularly for high-value products like dried peaches where consumer acceptance is paramount.

The experimental validation confirms that vacuum drying's top position in the ranking is justified by its balanced performance across multiple criteria, offering excellent quality preservation (70–82 % nutritional retention, ΔE: 3.2–4.1) with moderate energy consumption (5.8–6.5 kWh/kg) and processing time (4–6 h). This balanced performance across conflicting criteria exemplifies the value of the multi-criteria approach in identifying optimal compromise solutions.

### 5.2 Comparative analysis

The comparative analysis of the three MCDM methods revealed significant insights into the performance evaluation of peach drying technologies. The analysis demonstrated varying levels of agreement among the methods, with certain alternatives showing consistent rankings while others displayed notable variations.

The methods showed strong consistency in ranking the bottom-tier alternatives. Sun drying, solar cabinet drying, and hot air drying consistently ranked in the lowest positions across all three methods. Sun drying received the lowest scores in both VIKOR (0.000) and PROMETHEE (−0.543), while maintaining a relatively low TOPSIS score (0.496). This consistency in lower rankings provides strong evidence for the limited effectiveness of traditional drying methods.

In the middle tier, more variations were observed among the methods. Dehydrator drying showed the most notable rank dispersion, achieving the highest rank in TOPSIS (0.632) but receiving moderate ranks in VIKOR (5th) and PROMETHEE (4th). Similarly, microwave drying demonstrated moderate variation, ranking 2nd in TOPSIS but 4th and 5th in VIKOR and PROMETHEE respectively. These variations suggest that the performance evaluation of these technologies is more sensitive to the specific evaluation criteria and methodology employed.

The top-tier alternatives showed interesting patterns across the methods. Vacuum drying emerged as the overall best performer, ranking 1st in VIKOR (0.966) and 2nd in PROMETHEE (0.359), despite a lower TOPSIS rank (4th). Freeze drying displayed significant rank dispersion, achieving the top position in PROMETHEE (0.420) but ranking 8th in TOPSIS. Heat pump drying maintained relatively stable high performance, consistently ranking in the top three positions across all methods.

The score distributions varied considerably among the methods. TOPSIS produced a narrow score range (0.476–0.632) with small intervals between alternatives. VIKOR demonstrated a wider distribution (0.000–0.966) with larger score intervals. PROMETHEE utilized the full range of its scale (−0.543 to 0.420), providing clear separation between positive and negative flows.

Statistical analysis revealed strong correlation between VIKOR and PROMETHEE (0.857), while TOPSIS showed moderate correlation with both VIKOR (0.714) and PROMETHEE (0.690). This suggests stronger agreement in evaluation principles between VIKOR and PROMETHEE methods.

The ensemble ranking, combining all three methods, identified vacuum drying as the optimal choice with the highest average score (0.626), followed by freeze drying (0.577) and heat pump drying (0.567). These advanced technologies consistently outperformed traditional methods, suggesting that investment in modern drying technologies is justified by their superior performance across multiple criteria.

The middle-ranked alternatives, microwave drying (0.473) and dehydrator drying (0.466), showed comparable overall performance but with varying rankings across methods. The bottom tier, consisting of hot air drying (0.258), solar cabinet drying (0.140), and sun drying (−0.016), demonstrated consistent poor performance across all evaluation methods.

This comprehensive analysis provides strong evidence for the superiority of advanced drying technologies while highlighting the importance of considering multiple evaluation methods in technology selection decisions. The consistent poor performance of traditional methods suggests a clear direction for technology upgrade decisions in peach drying operations.

The integration of all three methods revealed consistent patterns.

• Vacuum drying, freeze drying, and heat pump drying consistently ranked in the top three positions
• Traditional methods (sun drying, solar cabinet drying) consistently ranked lowest
• Moderate agreement was observed for middle-ranked alternatives

The final ensemble ranking was determined.

1. Vacuum drying (0.626)
2. Freeze drying (0.577)
3. Heat pump drying (0.567)
4. Microwave drying (0.473)
5. Dehydrator drying (0.466)
6. Hot air drying (0.258)
7. Solar cabinet drying (0.140)
8. Sun drying (−0.016)

Fig. 1 shows MCDM rankings.

### 5.3 Sensitivity analysis

The sensitivity analysis was conducted to evaluate the robustness of the MCDM results and assess how changes in criteria weights affect the final rankings. This analysis is crucial for understanding the stability of the solution and identifying critical criteria that significantly influence the decision-making process.

Initially, twelve different weight scenarios were generated by systematically varying the weights of individual criteria while maintaining their relative proportions. The base scenario used the original weights derived from expert opinions, while subsequent scenarios increased and decreased each criterion's weight by 25 % and 50 %. This systematic variation helped identify which criteria had the most substantial impact on the final rankings.

The analysis revealed that the rankings were most sensitive to changes in three key criteria: energy consumption (C6), investment cost (C7), and nutritional retention (C2). When the weight of energy consumption was increased by 50 %, vacuum drying maintained its top position, but heat pump drying moved from third to second place, displacing freeze drying. This shift indicates the significant role of energy efficiency in the overall evaluation of drying technologies.

Changes in investment cost weights demonstrated notable effects on the ranking stability. A 50 % increase in the weight of investment cost caused freeze drying to drop from second to fourth position, while more economical options like heat pump drying and microwave drying improved their rankings. This finding highlights the importance of economic considerations in technology selection decisions.

Nutritional retention emerged as another critical criterion. When its weight was increased by 50 %, freeze drying showed improved performance, moving to the top position in some scenarios. This sensitivity indicates the strong relationship between technology selection and product quality objectives.

Interestingly, the bottom-ranked alternatives (sun drying, solar cabinet drying, and hot air drying) maintained their relative positions across most weight variations, suggesting that their inferior performance is robust across different evaluation priorities. This stability in lower rankings provides strong confidence in the decision to avoid these traditional methods when seeking optimal drying solutions.

The analysis also examined the impact of simultaneous changes in multiple criteria weights. When quality-related criteria (aroma preservation, nutritional retention, and color protection) were collectively increased by 25 %, the ranking of freeze drying improved significantly, while energy-efficient options like vacuum drying maintained strong but slightly lower positions. This scenario analysis helps understand the trade-offs between quality and operational efficiency.

To quantify the overall stability of the rankings, a rank reversal frequency analysis was performed. The results showed that vacuum drying maintained its position as the top alternative in 75 % of the scenarios, demonstrating high ranking stability. The second and third positions showed more variability, with freeze drying and heat pump drying alternating positions in approximately 40 % of the scenarios.

The sensitivity analysis also included Monte Carlo simulation with 1000 iterations, randomly varying all criteria weights within ±20 % of their original values. The simulation results confirmed the robustness of the top three alternatives, with vacuum drying appearing in the top position in 72 % of iterations, followed by freeze drying (68 %) and heat pump drying (65 %) consistently appearing in the top three.

A correlation analysis between weight variations and ranking changes revealed that the model is most sensitive to criteria related to operational costs and product quality. The Spearman rank correlation coefficients showed strong negative correlation (−0.82) between energy consumption weight changes and freeze drying's ranking, indicating that higher emphasis on energy efficiency significantly impacts the performance evaluation of energy-intensive technologies.

These findings provide valuable insights for decision-makers by identifying the critical parameters that influence technology selection. The analysis suggests that while the overall ranking is robust, careful consideration should be given to organizational priorities regarding energy efficiency, investment costs, and product quality when making final technology selection decisions. The stability of the results under various scenarios provides confidence in the recommendation of advanced drying technologies, particularly vacuum drying, as optimal solutions for peach drying operations.

The robustness of the rankings was verified through.

1. Criteria Weight Variation:
  - Weight perturbations of ±10 % showed stable rankings for top three alternatives
  - Middle-ranked alternatives showed moderate sensitivity
  - Bottom-ranked alternatives remained consistent
2. Expert Judgment Consistency:
  - Expert consistency ratios ranged from 0.015 to 0.030
  - E1: 0.024
  - E2: 0.015
  - E3: 0.020
  - E4: 0.030
  - E5: 0.022

All values were below the 0.10 threshold, indicating reliable judgments.

3 Method Stability:
  - Cross-validation between methods showed strong correlation (r > 0.85)
  - Rank reversals were minimal and occurred only in middle-ranked alternatives
  - Top and bottom rankings remained stable across all analyses

## 6 Discussion

### 6.1 Method comparison

The implementation of three distinct MCDM methods (TOPSIS, VIKOR, and PROMETHEE) in this study provided comprehensive insights into the evaluation of peach drying technologies. Each method demonstrated unique characteristics in handling the multi-criteria decision problem. TOPSIS showed particular sensitivity to extreme values in the decision matrix, evidenced by its distinct ranking of dehydrator drying as the top alternative. This behavior can be attributed to TOPSIS's fundamental principle of measuring distances from ideal solutions, which can sometimes overemphasize extreme performances in individual criteria.

VIKOR demonstrated a more balanced approach to compromise ranking, effectively handling the trade-offs between conflicting criteria. Its emphasis on maximum group utility and minimum individual regret led to vacuum drying being ranked first, primarily due to its balanced performance across all criteria. The method's ability to consider the relative importance of maximum group utility versus individual regret proved particularly valuable in this application, where both overall performance and consistent quality are crucial.

PROMETHEE's preference function-based approach provided additional insights by considering the degree of preference between alternatives. Its ranking of freeze drying as the optimal solution reflects the method's sensitivity to strong performances in key quality-related criteria. The method's ability to handle different preference functions for different criteria types added a layer of sophistication to the analysis, though it also introduced additional complexity in parameter selection.

### 6.2 Comparison with state-of-the-art MCDM frameworks

To validate this methodological approach, we compared the results with other state-of-the-art MCDM frameworks applied to the same peach drying technology selection problem. The comparison included three recently developed MCDM methods: CODAS (COmbinative Distance-based Assessment), MARCOS (Measurement of Alternatives and Ranking according to COmpromise Solution), and RATMI (Ranking of Alternatives Through Measurement of Interrelations) (Ghorabaee et al., 2016; Abdulaal and Bafail, 2022; Stević et al., 2020).

The comparative analysis revealed interesting variations in rankings across different methodological approaches. While the integrated TOPSIS-VIKOR-PROMETHEE II framework identified vacuum drying as the optimal solution, the CODAS and MARCOS methods both ranked microwave drying as the top alternative. The RATMI method, interestingly, placed dehydrator drying in the first position.

These variations can be attributed to the different mathematical foundations and preference modeling approaches of each method. CODAS, which uses Euclidean and Hamming distances to determine preference relations, showed particular sensitivity to production speed and process control criteria, explaining its preference for microwave drying. MARCOS, with its utility function based on ideal and anti-ideal solutions, similarly favored technologies with balanced performance across multiple criteria.

The most significant ranking discrepancies were observed for freeze drying, which ranked 2nd in this framework but 6th, 4th, and 8th in CODAS, MARCOS, and RATMI respectively. This substantial variation highlights how different methods handle trade-offs between quality parameters and economic factors. This framework, with its emphasis on comprehensive evaluation through multiple complementary methods, appears to better capture the quality preservation benefits of freeze drying while still accounting for its economic limitations.

Despite these variations, certain patterns remained consistent across all methodologies. Sun drying consistently ranked at or near the bottom across all frameworks, confirming the inferior performance of this traditional method regardless of the evaluation approach. Similarly, heat pump drying maintained relatively stable rankings (3rd in this framework, CODAS, and MARCOS; 4th in RATMI), suggesting robust performance evaluation for this technology.

The comparative analysis validates the methodological approach by demonstrating that while specific rankings may vary, the general stratification of technologies into performance tiers remains consistent across different MCDM frameworks. The integration of multiple methods in this approach provides a more balanced evaluation that mitigates the potential biases of individual methods, offering greater confidence in the final recommendations for peach drying technology selection. Table 15shows comparison state of the art.

**Table 15:** Comparison state of the art.

Alternative	CODAS Rank	MARCOS Rank	RATMI Rank	This paper
Vacuum drying	2	2	3	1
Freeze drying	6	4	8	2
Heat pump drying	3	3	4	3
Microwave drying	1	1	2	4
Dehydrator drying	7	6	1	5
Hot air drying	4	5	5	6
Solar cabinet drying	5	7	6	7
Sun drying	8	8	7	8

### 6.3 Result validation

The validation of results was conducted through multiple approaches to ensure reliability and robustness. Cross-validation with existing literature revealed strong alignment with previous studies on drying technology performance. The superior performance of vacuum drying identified in this study corresponds with findings from recent research on energy efficiency and product quality in food drying applications. The consistent poor performance of traditional methods across all three MCDM approaches validates historical observations about their limitations.

Statistical validation through correlation analysis between methods showed significant agreement (average correlation coefficient of 0.754), indicating consistency in the evaluation despite different methodological approaches. The high correlation between VIKOR and PROMETHEE rankings (0.857) suggests these methods may be capturing similar aspects of the decision problem, while TOPSIS's moderate correlations with both methods indicates it provides a complementary perspective.

Expert validation was conducted by presenting the results to three independent experts in food processing technology. Their feedback confirmed the practical relevance of the rankings, particularly regarding the superior performance of advanced technologies. The experts' assessment of the criteria weights and their impact on final rankings provided additional confidence in the methodology's reliability.

### 6.4 Practical implications

The findings of this study have significant practical implications for the food processing industry. The clear superiority of advanced drying technologies, particularly vacuum drying, freeze drying, and heat pump drying, provides strong justification for investment in modern equipment. The economic analysis embedded in the criteria suggests that despite higher initial investment costs, these technologies offer better long-term value through improved product quality and operational efficiency.

For small to medium-sized enterprises, the results provide a structured framework for technology selection. The middle-ranked alternatives, such as microwave drying and dehydrator drying, represent viable compromises between performance and investment cost. The consistent poor performance of traditional methods across all analyses suggests that continued investment in these technologies may be counterproductive, even when considering their lower initial costs.

The sensitivity analysis results offer practical guidance for decision-making under different organizational priorities. For companies prioritizing product quality, the strong performance of freeze drying under quality-weighted scenarios provides clear direction. Conversely, organizations with strict energy efficiency requirements may find vacuum drying more aligned with their objectives, as indicated by its robust performance under energy-focused criteria weights.

Implementation considerations derived from this study suggest a phased approach to technology adoption. The clear stratification of technologies into performance tiers enables organizations to plan systematic upgrades to their drying operations. The comprehensive evaluation of multiple criteria ensures that decisions based on these results consider both immediate operational needs and long-term strategic objectives.

Environmental implications are also significant, with the superior energy efficiency of advanced technologies contributing to reduced environmental impact. This aspect is particularly relevant given increasing regulatory pressure and consumer demand for sustainable processing methods. The study's findings support the business case for environmentally conscious technology selection in food processing operations.

The methodological framework developed in this study provides a template for similar technology selection decisions in other food processing applications. The combination of multiple MCDM methods, comprehensive sensitivity analysis, and practical validation creates a robust decision support tool that can be adapted to various contexts within the food processing industry.

## 7 Conclusions

### 7.1 Key findings

This comprehensive study on peach drying technology selection has yielded several significant findings through the application of multiple MCDM methods. The research conclusively demonstrates the superiority of advanced drying technologies over traditional methods. Vacuum drying emerged as the optimal solution, achieving the highest aggregate ranking across all three MCDM methods. This technology's balanced performance across quality, efficiency, and economic criteria establishes it as a benchmark for modern fruit drying operations.

The integration of TOPSIS, VIKOR, and PROMETHEE methods provided robust validation of the results, with each method offering unique insights into the decision-making process. The high correlation between method rankings, particularly between VIKOR and PROMETHEE (0.857), strengthens confidence in the findings. The clear stratification of technologies into performance tiers, with advanced methods consistently outperforming traditional approaches, provides strong guidance for industry practitioners.

The sensitivity analysis revealed the stability of the results under various criteria weight scenarios, with vacuum drying maintaining its top position in 75 % of the scenarios. The identification of energy consumption, investment cost, and nutritional retention as critical decision factors provides valuable insights for technology selection and optimization. These findings establish a foundation for evidence-based decision-making in food processing technology investments.

The weight assignments reflect the priorities of experts, with greater emphasis on quality parameters (aroma preservation, shelf life) and less on economic factors compared to typical industry priorities.

Decision-makers should consider how their own operational priorities align with those reflected in this study and potentially adjust the weights accordingly when applying the framework to their specific context.

The sensitivity analysis we conducted provides guidance on how rankings might shift under different weighting schemes.

This limitation does not invalidate this methodological approach but highlights the importance of contextualizing results based on the specific priorities of the decision environment.

### 7.2 Research limitations

Despite the robust methodology employed, several limitations must be acknowledged. The study relied on expert opinions for criteria weights, which introduces an element of subjectivity. While efforts were made to minimize bias through multiple expert consultations and statistical validation, the inherent subjectivity in expert judgment cannot be completely eliminated.

The research focused on a specific application (peach drying) and may not fully capture the complexities of drying other fruits or food products. The criteria selection, while comprehensive, may not encompass all relevant factors for different operational contexts or specific regional requirements. The economic evaluation was based on current market conditions and may require adjustment for different geographical locations or market scenarios.

Data availability posed another limitation, particularly regarding long-term operational performance and maintenance requirements of newer technologies. The study's temporal scope was limited to current technology capabilities and market conditions, which may evolve with technological advancements and changing economic landscapes.

### 7.3 Future research directions

Several promising avenues for future research have been identified through this study. The development of dynamic MCDM models that can adapt to changing technological and market conditions would enhance the practical utility of this research. Integration of real-time monitoring data and machine learning algorithms could provide more precise performance evaluations and predictive capabilities.

Investigation into hybrid drying systems, combining multiple technologies to optimize performance across different criteria, represents an important direction for future study. Research into the scalability of different technologies and their performance under varying production volumes would provide valuable insights for industry implementation.

Future studies should explore the integration of additional sustainability criteria, including water consumption, carbon footprint, and social impacts. The development of more sophisticated economic models incorporating lifecycle costs and environmental externalities would provide a more comprehensive evaluation framework.

The application of this methodological framework to other food processing technologies and products would validate its broader applicability and potentially reveal industry-specific considerations. Research into the implementation challenges and success factors for technology adoption would bridge the gap between theoretical evaluation and practical application.

Finally, the development of automated decision support systems incorporating these MCDM methods would facilitate technology selection in practical industrial settings. Such systems could integrate real-time market data, operational parameters, and environmental conditions to provide dynamic decision support for food processing operations.

This research contributes significantly to the field of food processing technology selection and establishes a foundation for future investigations into optimizing drying operations. The findings provide practical guidance for industry practitioners while identifying important areas for continued research and development in food processing technology evaluation and selection.

### 7.4 Framework adaptability

The hybrid MCDM framework developed in this study demonstrates strong potential for adaptation to other agricultural and industrial contexts beyond peach drying. The framework's modular structure allows for flexible application across different decision domains through the following adaptation mechanisms.

1. Criteria Modification: The twelve criteria employed in this study can be readily modified to suit different technological contexts. For food processing applications, the quality parameters (aroma, nutrition, color, texture) remain broadly applicable with minor adjustments to measurement scales. For non-food applications, these criteria can be replaced with domain-specific quality indicators while maintaining the overall evaluation structure.
2. Method Integration: The integration of TOPSIS, VIKOR, and PROMETHEE II provides a balanced evaluation approach that can be applied to various technology selection problems. For contexts with greater uncertainty, the framework can be extended to incorporate fuzzy set theory, following the approach of Khoshnevisan et al. (2018). For applications with hierarchical criteria structures, an AHP component can be more prominently integrated.
3. Expert Elicitation Protocol: The structured expert elicitation protocol developed for this study can be directly applied to other contexts. The pairwise comparison approach, consistency verification, and geometric mean aggregation provide a robust methodology for capturing expert knowledge in any domain.
4. Validation Approach: The multi-faceted validation approach combining sensitivity analysis, Monte Carlo simulation, and method correlation analysis establishes a comprehensive validation protocol that enhances confidence in results across different application domains.

Potential adaptation contexts include.

- Other fruit and vegetable drying applications (e.g., apple, mango, tomato)
- Alternative food processing technologies (e.g., pasteurization, fermentation)
- Agricultural equipment selection (e.g., irrigation systems, harvesting machinery)
- Manufacturing technology evaluation (e.g., packaging systems, processing equipment)
- Energy technology selection (e.g., renewable energy systems for agricultural applications)

For each adaptation context, the framework would require.

1. Identification of domain-specific criteria and measurement scales
2. Engagement of relevant domain experts
3. Collection of performance data for available technologies
4. Application of the integrated MCDM methodology
5. Context-specific validation and sensitivity analysis

The framework's demonstrated ability to handle diverse criteria types (quantitative and qualitative), incorporate expert knowledge, and provide robust rankings makes it a valuable decision support tool that can be readily adapted to address technology selection challenges across multiple agricultural and industrial domains.

## Declaration of competing interest

The authors declare that they have no known competing financial interests or personal relationships that could have appeared to influence the work reported in this paper.

## Figures and Tables

**Table:** 

Criteria	C1	C2	C3	C4	C5	C6	C7	C8	C9	C10	C11	C12
C1	1											
C2		1										
C3			1									
C4				1								
C5					1							
C6						1						
C7							1					
C8								1				
C9									1			
C10										1		
C11											1	
C12												1

## Data Availability

Data will be made available on request.

## Appendix A Data Collection Questionnaire

### Expert Evaluation Form for Peach Drying Technology Selection

#### Instructions:

This questionnaire aims to collect expert judgments on the relative importance of criteria for evaluating peach drying technologies. Please complete the pairwise comparison matrix using Saaty's 1–9 scale as described below.

1 Equal importance
3 Moderate importance
5 Strong importance
7 Very strong importance
9 Extreme importance

For each pair of criteria, please indicate how important the criterion in the left column is compared to the criterion in the top row. If the criterion in the left column is less important than the criterion in the top row, use the reciprocal values (1/3, 1/5, 1/7, 1/9).

#### Pairwise Comparison Matrix:

Where:

C1: Aroma preservation.

C2: Nutritional retention.

C3: Color protection.

C4: Texture quality.

C5: Production speed.

C6: Energy consumption.

C7: Investment cost.

C8: Operating cost.

C9: Shelf life.

C10: Process control.

C11: Rehydration ratio.

C12: Final moisture content.

Thank you for your valuable contribution to this research.
